# Complete Genome Sequence of Enterococcus faecium FS86, Used for Propagation of Bacteriophages with Therapeutic Potential

**DOI:** 10.1128/MRA.00776-20

**Published:** 2020-09-24

**Authors:** Rustam M. Buzikov, Emma G. Piligrimova, Andrey M. Shadrin

**Affiliations:** a Laboratory of Bacteriophage Biology, G. K. Skryabin Institute of Biochemistry and Physiology of Microorganisms, Pushchino Scientific Center for Biological Research of the Russian Academy of Sciences, Federal Research Center, Pushchino, Moscow Oblast, Russia; Portland State University

## Abstract

The Enterococcus faecium FS86 genome consists of a 2,685,395-bp chromosome and a 9,751-bp plasmid. The plasmid harbors mobilization-related genes. The pathogenicity factor genotype is *cylA* negative, *aggA* negative, *gelE* negative, *sprE* negative, *esp* negative, *eep* positive, and *efaA* positive. E. faecium FS86 belongs to multilocus sequence type 296, together with the probiotic strain E. faecium SF68.

## ANNOUNCEMENT

Enterococcus faecium is a common member of the human gut microflora (1) that is often used as a probiotic (2). However, several strains of enterococci are known to be associated with urinary tract infections, bacteremia, endocarditis, and other infectious diseases (1). Bacteriophages can be used to treat infections caused by these antibiotic-resistant E. faecium strains (3). In this paper, we report the whole-genome sequence of the E. faecium FS86 strain, which was used for propagation of certain bacteriophages with therapeutic potential.

E. faecium FS86 was isolated from a capsule of Bifiform (Ferrosan) by plating the material onto KF *Streptococcus* agar (Difco). The procedure was repeated three times, and a single colony from the third plate was grown in a test tube with KF *Streptococcus* broth (Difco) to obtain a stock culture, which was maintained at −80°C. Total DNA was isolated by the standard phenol-chloroform DNA extraction method (4). The MiSeq library was prepared with the KAPA HyperPlus kit. A total of 29,676,276 Illumina MiSeq paired-end reads with an average length of 101 nucleotides (nt) were obtained. The Oxford Nanopore library was prepared using SQK-LSK109 and EXP-NBD104 kits; 19,280 Oxford Nanopore reads, with an average length of 2,374 nt (maximum read length, 77,526 nt), were used for the assembly. Genome assembly was performed using the Genome Assembly Service at the PATRIC v3.6.5 website (http://patricbrc.org) (5), with both Illumina paired-end reads and Nanopore reads, using the Unicycler v0.4.8 assembly strategy; read trimming was performed with the Trim Galore tool (6) implemented in PATRIC. The resulting assembly graph was visualized in Bandage v0.8.1 (7). All genome assembly software was used with default parameters, except that the “trim reads before assembly” parameter was changed to “true” for the Trim Galore tool. Two circular contigs (lengths of 2,685,395 bp and 9,751 bp, and average coverages of 751.5× and 3,006×, respectively) were obtained, suggesting that the genome is represented by a chromosome and a plasmid with a copy number of ∼4 per chromosome. The GC contents are 38.4% and 32.6% for the chromosome and the plasmid, respectively.

The E. faecium FS86 chromosome was annotated by the NCBI Prokaryotic Genome Annotation Pipeline v4.11 (8). In total, 2,594 putative genes were identified, including 2,503 coding sequences (CDSs), 6 clusters of the 16S, 23S, and 5S rRNA genes, 69 tRNA genes, 1 transfer-messenger RNA gene, and 3 noncoding RNA genes. The pFS86 plasmid was annotated by RAST v2 (9), and 14 genes were identified. The plasmid harbors mobilization (CDS1 to CDS3) and replication (CDS6 and CDS7) gene modules similar to those of the pHY (10) and pCIZ2 (11) plasmids harboring bacteriocin genes. No genes encoding antibiotic-modifying enzymes were detected on the plasmid, but the FS86 chromosome encodes aminoglycoside *N*-acetyltransferase AAC(6′)-Ii, providing resistance to aminoglycosides.

The FS86 strain belongs to sequence type 296 (ST296) from clonal cluster 98 (CC98) according to the scheme developed by Homan et al. (12, 13). Interestingly, the E. faecium SF68 strain, which has been widely used as a probiotic in the past 4 decades (2), also belongs to ST296.

### Data availability.

This whole-genome project has been deposited in GenBank under accession number CP053704 for the chromosome and accession number MT501398.1 for the plasmid. Raw reads are available in the Sequence Read Archive under BioProject accession number PRJNA633747.
